# Loss of NSD2 causes dysregulation of synaptic genes and altered H3K36 dimethylation in mice

**DOI:** 10.3389/fgene.2024.1308234

**Published:** 2024-02-14

**Authors:** Shiori Kinoshita, Kazuaki Kojima, Eriko Ohnishi, Yuka Takayama, Hiroki Kikuchi, Shuji Takada, Kazuhiko Nakabayashi, Tomoko Kawai, Kenichiro Hata

**Affiliations:** ^1^ Department of Maternal-Fetal Biology, National Research Institute for Child Health and Development, Tokyo, Japan; ^2^ Department of NCCHD Child Health and Development, Graduate School of Medical and Dental Sciences, Tokyo Medical and Dental University, Tokyo, Japan; ^3^ Department of Systems BioMedicine, National Research Institute for Child Health and Development, Tokyo, Japan; ^4^ Department of Human Molecular Genetics, Graduate School of Medicine, Gunma University, Maebashi, Gunma, Japan

**Keywords:** NSD2, neurodevelopmental disorder, RNA sequencing, ChIP sequencing, H3K36me2

## Abstract

**Background:** Epigenetic disruptions have been implicated in neurodevelopmental disorders. NSD2 is associated with developmental delay/intellectual disability; however, its role in brain development and function remains unclear.

**Methods:** We performed transcriptomic and epigenetic analyses using *Nsd2* knockout mice to better understand the role of NSD2 in the brain.

**Results and discussion:** Transcriptomic analysis revealed that the loss of NSD2 caused dysregulation of genes related to synaptic transmission and formation. By analyzing changes in H3 lysine 36 dimethylation (H3K36me2), NSD2-mediated H3K36me2 mainly marked quiescent state regions and the redistribution of H3K36me2 occurred at transcribed genes and enhancers. By integrating transcriptomic and epigenetic data, we observed that H3K36me2 changes in a subset of dysregulated genes related to synaptic transmission and formation. These results suggest that NSD2 is involved in the regulation of genes important for neural function through H3K36me2. Our findings provide insights into the role of NSD2 and improve our understanding of epigenetic regulation in the brain.

## 1 Introduction

Epigenetic mechanisms, including DNA methylation and histone post-translational modifications, guide spatiotemporal gene expression patterns in cells and play critical roles in many biological processes. Disruption of epigenetic regulation has been implicated in human diseases, including cancer and developmental disorders (Greenberg and Bourc’his, 2019; Husmann and Gozani, 2019). With recent advances in DNA sequencing technologies, the number of variants and genes associated with the risk and etiology of neurodevelopmental disorders (NDDs), including autism spectrum disorder (ASD) and global developmental delay/intellectual disability (DD/ID), has increased (Gilissen et al., 2014; Stessman et al., 2017; Coe et al., 2019; Satterstrom et al., 2020). In addition, variants in genes encoding the epigenetic machinery are linked to NDDs (Fahrner and Bjornsson, 2019), suggesting that epigenetic regulation and its disruption are highly relevant to the etiology of NDDs and brain development.

NSD2 (also known as MMSET or WHSC1) is a member of the nuclear receptor-binding SET domain family that mediates histone H3 lysine 36 mono- and dimethylation (H3K36me2) (Kuo et al., 2011). Variable-sized deletions in chromosome 4p16.3, including NSD2, cause Wolf–Hirschhorn syndrome (also known as 4p-syndrome; OMIM#194190), which is characterized by growth retardation, DD/ID, microcephaly, hypotonia, and congenital malformations (Bergemann et al., 2005; Zollino et al., 2008; Battaglia et al., 2015). Recent human genetic studies have identified missense and truncating variants of NSD2 in individuals with DD/ID and growth retardation (known as Rauch–Steindl syndrome; OMIM#619695) (Zanoni et al., 2021). Most missense variants impair NSD2 enzymatic activity to mediate H3K36me2. This suggests that loss of NSD2 function could lead to these clinical manifestations. NSD2 plays critical roles in normal development, including cardiac development, adipose tissue development and function, follicular helper T-cell differentiation, and B-cell development (Nimura et al., 2009; Campos-Sanchez et al., 2017; Zhuang et al., 2018; Long et al., 2020); however, its function in brain development remains unclear.

In this study, we characterized the effects of loss of NSD2 on transcriptional and epigenetic landscapes in the brain using knockout (KO) mice to better understand its role in brain development and function.

## 2 Materials and methods

### 2.1 Mice

*Nsd2* KO mice were generated using CRISPR/Cas9 genome editing, as described previously (Kawai et al., 2023). All mice used in this study were maintained on the C57BL/6 background. All animal experiments were conducted following the protocol approved by the Animal Care and Use Committee of the National Research Institute for Child Health and Development, Tokyo, Japan (Permit No. A2016-001).

### 2.2 Protein extraction and western blotting

Proteins were extracted from the embryonic brain as described previously (Oliviero et al., 2016) with slight modifications. The embryonic brain was homogenized in a lysis buffer (25 mM Tris-HCl [pH 7.6], 150 mM NaCl, 1% NP-40) with protease inhibitors (Nacalai Tesque, Kyoto, Japan). The lysates were incubated for 15 min on ice and centrifuged at 18,000 × *g* for 15 min at 4°C. To harvest the nuclear fraction, nuclear pellets were resuspended in high salt containing Buffer C (20 mM HEPES [pH 7.6], 20% [v/v] glycerol, 0.42 M NaCl, 1.5 mM MgCl_2_, 0.2 mM EDTA) with Benzonase nuclease (Merck KGaA, Darmstadt, Germany) and dounced 20 times with a homogenizer. Lysates were incubated for 45 min with rotation and centrifuged at 18,000 × *g* for 30 min at 4 °C. Before loading, Laemmli sample buffer with β-mercaptoethanol (Bio-Rad, Hercules, CA, United States) was added to the samples, followed by boiling at 95 °C for 5 min. Protein samples were resolved using sodium dodecyl sulfate-polyacrylamide gel electrophoresis, transferred onto a polyvinylidene fluoride membrane (Bio-Rad), blocked in 2% BSA in TBS-T or Blocking One (Nacalai Tesque), probed with primary antibodies, and detected using horseradish peroxidase-conjugated anti-rabbit or anti-mouse secondary antibodies (GE Healthcare, Chicago, IL, United States). Primary antibodies used in this study included anti-Histone H3 (ab1791, Abcam, Cambridge, United Kingdom), anti-NSD2 (ab75359, Abcam), andanti-H3K36me2 (ab9049, Abcam). Chemiluminescence was detected using ImageQuant LAS 4000 (GE Healthcare). Quantification was performed using ImageJ (Schneider et al., 2012).

### 2.3 Tissue fixation, sectioning, and staining

Embryonic brains from E18.5 were dissected and fixed by immersion in a 10% formalin neutral buffer solution (FUJIFILM Wako Pure Chemical Corporation, Osaka, Japan). The brains were then processed using an automated tissue processor (ASP 200, Leica Biosystems, Wetzlar, Germany), embedded in paraffin, and sectioned at a thickness of 5 μm on a rotary microtome. The sections were stained with 0.1% cresyl violet (Muto Pure Chemicals, Tokyo, Japan).

### 2.4 RNA sequencing (RNA-seq)

RNA-seq analysis was performed for the E15.5 brains of WT (n = 3; female = 3, male = 3) and *Nsd2* KO mice (n = 3; female = 3, male = 3). Total RNA was extracted from E15.5 brains using the AllPrep DNA/RNA Mini Kit (Qiagen, Hilden, Germany). Total RNA purity and integrity were assessed using the BioAnalyzer RNA Pico kit (Agilent Technologies, Santa Clara, CA, United States). Samples with an RNA integrity number ≥7 were used for library preparation. Ribosomal RNA was depleted using the NEBNext rRNA Depletion Kit or Kit v2 (Human/Mouse/Rat) (New England Biolabs, Ipswich, MA, United States), and cDNA libraries were generated using the NEBNext Ultra II Directional RNA Library Prep Kit for Illumina (New England Biolabs) following the manufacturer’s protocol. The libraries were sequenced on either the HiSeq X platform (Illumina, San Diego, CA, United States), generating 150 bp paired-end reads or the NextSeq 550 platform (Illumina), generating 75 bp paired-end reads.

### 2.5 RNA-seq data processing and analysis

All 150 bp paired-end reads were trimmed to a length of 75 bp using FASTX-toolkit (v0.0.14)^1^ to ensure the same analytical conditions as the 75 bp paired-end reads. Reads containing adapter sequences were trimmed using cutadapt (v.2.6) (Martin, 2011). The reads were aligned to the mouse genome (mm10) using HISAT2 (v.2.1.0) (Kim et al., 2019). Transcripts were assembled and quantified using StringTie (v. 2.0.6) (Pertea et al., 2015). For analysis of RNA-seq data, all transcripts from mouse Ensembl genes 81 version (GRCm38.p4/mm10) were used. The gene counts matrix was generated by the Python script “prepDE.py” to extract read count information from StringTie output. Differential expression analysis was performed using the edgeR package (Robinson et al., 2010), and DEGs were defined using a false discovery rate cutoff of 0.05. Gene Ontology (GO) and Kyoto Encyclopedia of Genes and Genomes (KEGG) pathway enrichment analyses were performed using the Metascape software (Zhou et al., 2019).

### 2.6 Quantitative reverse transcription PCR (qRT-PCR)

RNA extraction was performed as described above in section 2.4. cDNA was synthesized using PrimeScript RT reagent Kit with gDNA Eraser (Perfect Real Time) (Takara Bio, Shiga, Japan). PCR was performed on the Applied Biosystems 7,500 Fast Real-Time PCR System (Thermo Fisher Scientific, Waltham, MA, United States) using TB Green Premix Ex TaqⅡ (Tli RNaseH Plus) (Takara Bio) with primers listed in Supplementary Table S1. Relative expression was calculated using the ∆∆Ct method and data were normalized to *Actb* expression.

### 2.7 Chromatin immunoprecipitation followed by DNA sequencing (ChIP-seq) and ChIP-qPCR

ChIP-seq analysis was performed for the E15.5 brains of WT and *Nsd2* KO mice (n = 2; male = 2 in each group). ChIP experiments were performed using ChIP Reagents (Nippon Gene, Tokyo, Japan). ChIP assay was performed on E15.5 WT and *Nsd2* KO brains. Fresh brain tissues were minced, fixed with 1% paraformaldehyde in D-PBS (−) for 10 min, and quenched with 2 M glycine for 5 min. The tissues were resuspended in NP-40 buffer containing a proteinase inhibitor cocktail (Nacalai Tesque) and homogenized using BioMAsher (Nippi, Tokyo, Japan). The homogenized tissue was incubated on ice for 10 min and centrifuged to collect the nuclear pellet. The nuclear pellet was resuspended in SDS Lysis buffer, and the lysate was sonicated to fragment the chromatin for 15 min (duty: 5, PIP: 105, cycles/burst: 200) on Covaris S220 (Covaris, Woburn, MA, United States). The fragmented chromatin was centrifuged to remove debris and immunoprecipitated with Dynabeads Protein A (Thermo Fisher Scientific)-conjugated anti-H3K36me2 antibodies (ab9049, Abcam) or normal Rabbit IgG (#2729, Cell Signaling Technology, Danvers, MA, United States) in ChIP dilution buffer containing protease inhibitor cocktail (Nacalai Tesque) for 4 h at 4°C with rotation. The beads were washed with 1× RIPA buffer (150 mM, 1× RIPA buffer (500 mM), LiCl buffer (250 mM LiCl, 10 mM Tris-HCl [pH 8.0], 0.5% NP-40, 0.5% sodium deoxycholate, and 1 mM EDTA), and TE buffer (pH 8.0). The beads were incubated in ChIP direct elution buffer with Proteinase K overnight at 65°C. ChIP DNA was purified using AMPure XP beads (Beckman Coulter, Brea, CA, United States) following the manufacturer’s protocol. For ChIP-seq, libraries were generated using the NEBNext Ultra II DNA Library Prep Kit for Illumina (New England Biolabs) following the manufacturer’s protocol. The libraries were sequenced on a NextSeq550 platform (Illumina) with 75 bp single-end and paired-end reads. ChIP-qPCR was performed on Applied Biosystems 7500 Fast Real-Time PCR System (Thermo Fisher Scientific) using TB Green Premix Ex TaqⅡ (Tli RNaseH Plus) (Takara Bio) with primers listed in Supplementary Table S2.

### 2.8 ChIP-seq data processing and analysis

The adapter trimming method was the same as that used for RNA-seq analysis. The ChIP-seq reads were aligned to the mouse genome (mm10) using BWA-MEM (v.0.7.17) (Li and Durbin, 2009). PCR duplicates were removed using Picard (v.2.17.11)^2^, and bam files of each biological replicate were merged using SAMtools (v.1.6) (Danecek et al., 2021). Bigwig tracks were generated using bamCoverage in deepTools (v.3.3.0) with options “--normalizeUsing CPM.” To calculate normalized H3K36me2 ChIP signal for each window (1 kb windows, 10 k windows), read counts were divided by input read counts using bamCompare in deepTools (v.3.3.0) (Ramírez et al., 2014) with option “--normalizeUsing CPM, -- operation ratio.” Fold change (FC) of normalized H3K36me2 ChIP- signal (10 k windows) was used to identify H3K36me2 loss or gain at thresholds of FC < 1/1.5 (loss) or >1.5 (gain). The correlation map was presented using computeMatrix and plotCorrelation in deepTools (v.3.3.0) (Ramírez et al., 2014). The pairwise correlation (Spearman’s ρ) coefficient was determined using the normalized H3K36me2 ChIP signal in 10 k windows. The blacklisted regions downloaded from the ENCODE portal (ENCFF547MET) (Amemiya et al., 2019; Luo et al., 2020) were excluded from the downstream analysis. The distribution of H3K36me2 across the mouse genome was determined using ChIPseeker (v.1.34.1) (Yu et al., 2015). Random 10 kb windows were generated using BEDTools (v. 2.26.0) (Quinlan and Hall, 2010). ChromHMM annotation-based enrichment analysis was performed using LOLA (v.1.28.0) (Sheffield and Bock, 2016) and the E15.5 mouse forebrain ChromHMM annotation downloaded from the ENCODE portal (ENCFF163AVC) (Luo et al., 2020; van der Velde et al., 2021). The heatmap and aggregation plot were depicted using computeMatrix, plotProfile, and plotHeatmap in deepTools (v.3.3.0) (Ramírez et al., 2014). For enhancer analysis, H3K4me1, H3K27ac, and H3K4me3 peak files of the E15.5 mouse forebrain were downloaded from the ENCODE portal (ENCFF871XIM, ENCFF433OHF, ENCFF112LTJ). Active enhancers were defined as both H3K4me1 and H3K27ac peaks without H3K4me3 peaks. Super enhancers were called from H3K27ac peak files (ENCFF433OHF) using ROSE (Whyte et al., 2013). Putative target genes active enhancer pairs were determined using GREAT (Tanigawa et al., 2022).

## 3 Results

### 3.1 Loss of NSD2 causes transcriptional dysregulation of synaptic transmission and formation-related genes in the brain

We previously generated an *Nsd2* KO mouse model in which the entire coding sequence of *Nsd2* was deleted (Supplementary Figure S1A) (Kawai et al., 2023). NSD2 protein expression was barely detectable in *Nsd2* KO mice (Supplementary Figure S1B). *Nsd2* KO mice exhibited lower body weight compared to wild-type (WT) mice and died within 1 day of birth (Supplementary Figure S1C).

On the basis of the high expression of *Nsd2* during the embryonic stage (Supplementary Figure S1D) and the known association between epigenetic regulation and brain development (Fagiolini et al., 2009), we focused on its potential role in brain development and function during this stage. First, we evaluated the brain morphology of *Nsd2* KO mice. The total brain weight of *Nsd2* KO mice was significantly reduced compared to WT mice. However, there was no significant difference in the brain/body weight ratio between WT and *Nsd2* KO mice (Supplementary Figure S1E), suggesting that the reduced brain weight was due to growth retardation. Although histological analysis of the brain did not reveal any apparent structural defects in *Nsd2* KO mice, the cortex of *Nsd2* KO mice showed a slight reduction in thickness compared to the control (Supplementary Figure S1F). Thus, loss of NSD2 has minimal effects on the cytoarchitecture of the brain during the embryonic stage.

**FIGURE 1:**
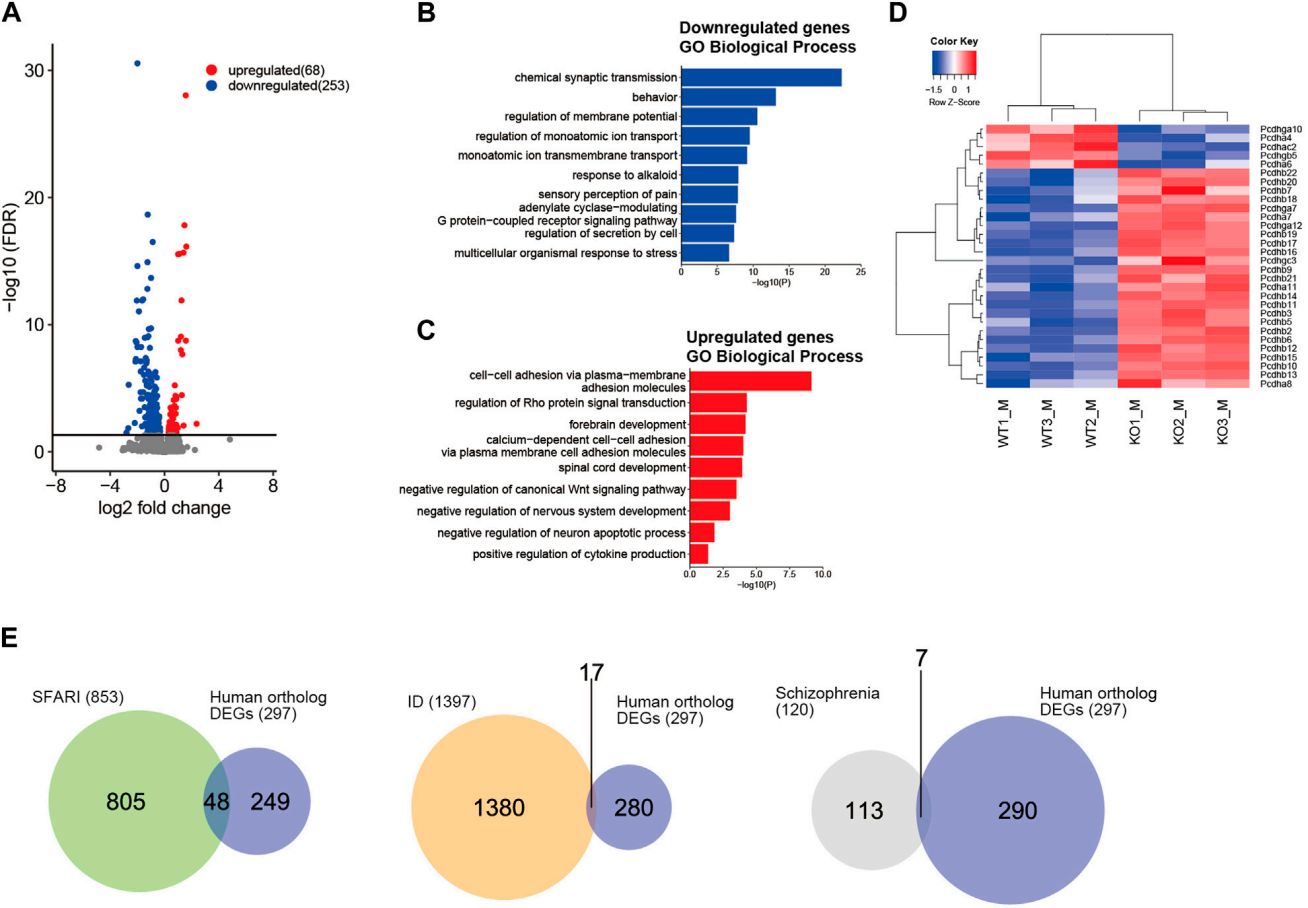
Loss of NSD2 affects gene expression in the brain. **(A)** Volcano plot of RNA sequencing analysis showing differentially expressed genes (DEGs) between *Nsd2* knockout (KO) and wild-type (WT) male mouse brains at E15.5; n = 3 in each group. DEGs are labeled in blue (downregulated) and red (upregulated). The horizontal line indicates the false discovery rate (FDR) threshold of 0.05. **(B, C)** Bar plots showing enriched Gene Ontology terms among biological processes related to downregulated DEGs **(B)** and upregulated DEGs **(C)** in *Nsd2* KO males (*p* < 0.05). **(D)** Heatmap showing clustered protocadherin genes differentially expressed in *Nsd2* KO males by hierarchical clustering of WT and *Nsd2* KO biological replicates. **(E)** Venn diagram showing the overlap between human ortholog DEGs in *Nsd2* KO male and neurodevelopmental disorder gene sets, including autism spectrum disorder from SFARI (left), intellectual disability (middle), and schizophrenia (right). ASD, autism spectrum disorder; ID, intellectual disability.

NSD2 mediates H3K36me2, which is generally associated with active transcription (Kuo et al., 2011; Weinberg et al., 2019). We next performed RNA-seq analysis for WT and *Nsd2* KO brains at embryonic day (E) 15.5 to examine the transcriptional effect of NSD2 loss. E15.5 was selected as the preferred developmental time point for our samples because it is a critical period for neurodevelopment. This includes important processes such as neurogenesis, division and differentiation of neural progenitor cells, migration, and relatively homogeneous populations of cells (Yao and Jin, 2014; La Manno et al., 2021). We identified 321 differentially expressed genes (DEGs) (excluding *Nsd2*). Out of these, 253 were significantly downregulated and 68 were upregulated in *Nsd2* KO male brains (Figure 1A and Supplementary Table S3). Sixty-two out of the 321 DEGs consistently showed differential expression in both male and female KO mice (Supplementary Figures S2A, 2B and Supplementary Table S4). GO analysis revealed that the downregulated DEGs were involved in synaptic transmission in terms of biological processes (Figure 1B and Supplementary Figure S2C). Among the genes annotated as cellular components, the downregulated DEGs were related to the synaptic membrane and ion channel complex (Supplementary Figure S2D). Furthermore, KEGG pathway analysis revealed an enrichment of neuronal synapse and signaling pathways, specifically glutamatergic synapse, serotonergic synapse, and cAMP signaling (Supplementary Figure S2E). In contrast, the upregulated DEGs showed an enrichment of cell-cell adhesion molecules (Figure 1C). Notably, the *Nsd2* KO showed an upregulation of protocadherin beta genes (*Pcdhb*) (Figure 1D and Supplementary Figure S2F). Clustered protocadherin genes are cell surface molecules expressed in the central nervous system. They play crucial roles in shaping optimal neural circuits and promoting neuronal diversity (Lefebvre et al., 2012; Hasegawa et al., 2016; Mountoufaris et al., 2017). To further evaluate the association between these DEGs and NDDs, we analyzed the overlap between the human ortholog DEGs and genes related to NDDs, such as ASD (SFARI score 1–3; 853 genes) (Banerjee-Basu and Packer, 2010), ID (1397 genes) (Ilyas et al., 2020), and schizophrenia (120 genes) (Trubetskoy et al., 2022). The DEGs in *Nsd2* KO males and females overlapped with 3.0% and 1.1% of the NDD-related gene sets, respectively (Figure 1E and Supplementary Figure S2G). These results demonstrate that the loss of NSD2 impairs gene expression associated with synaptic transmission and formation.

### 3.2 Loss of NSD2 causes global changes in H3K36me2 in the brain

**FIGURE 2:**
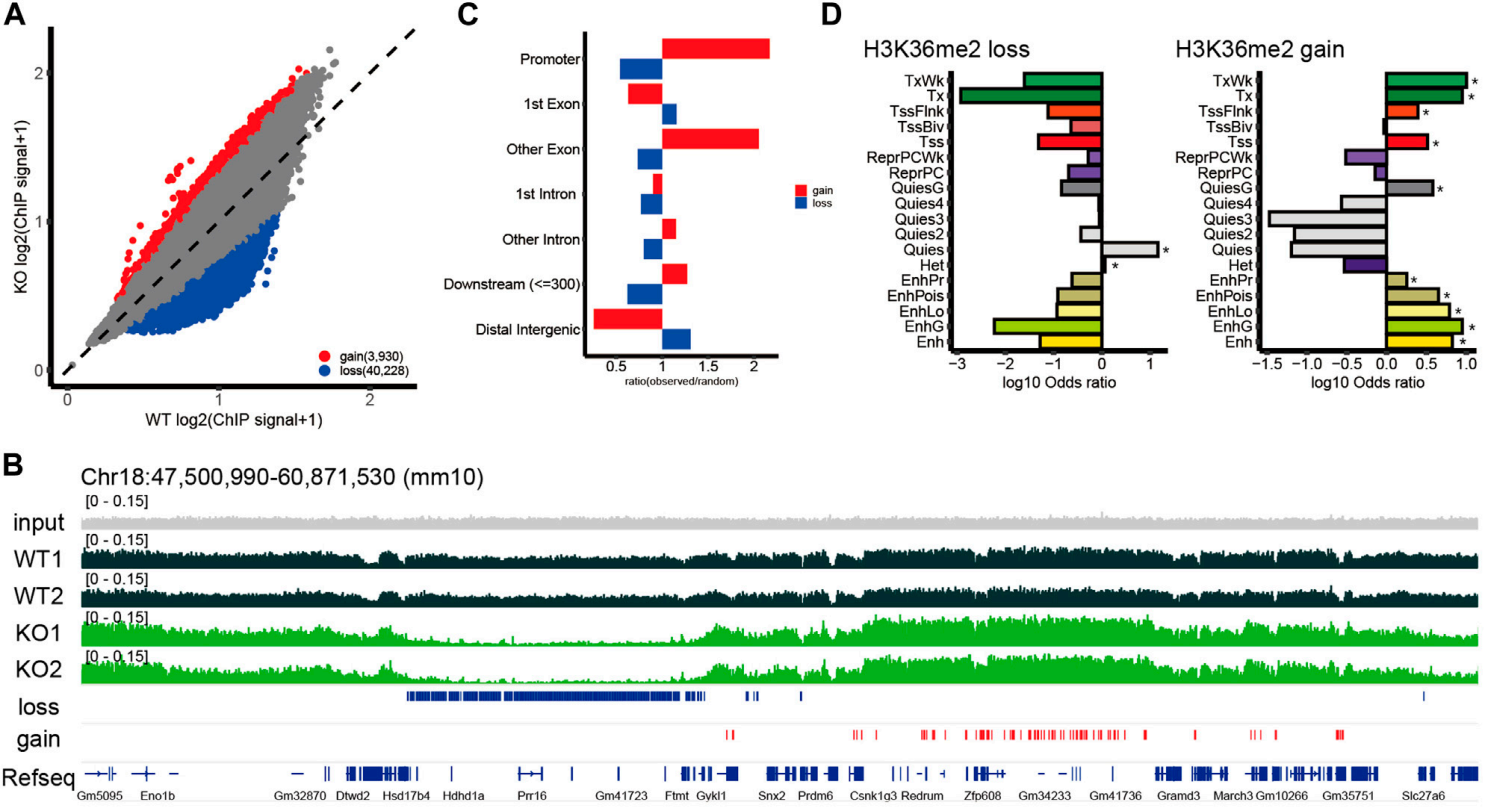
Genome-wide changes in H3K36me2. **(A)** Scatterplot showing normalized H3K36me2 chromatin immunoprecipitation (ChIP) signal for the 10 kb window in wild-type (WT) and *Nsd2* knockout (KO) E15.5 brains. The windows with loss or gain of H3K36me2 were defined as normalized H3K36me2 ChIP signal ratio between *Nsd2* KO *versus* WT E15.5 brain <1/1.5 or >1.5 and labeled in blue (loss) and red (gain). **(B)** Genome browser snapshot showing representative H3K36me2 ChIP signal in WT and *Nsd2* KO E15.5 brains. H3K36me2 loss/gain in *Nsd2* KO are shown as red and blue bars, respectively, and genes (from RefSeq) are annotated at the bottom. **(C)** Bar plot showing the ratio of observed-to-random H3K36me2 loss and gain in annotated genomic regions in *Nsd2* KO E15.5 brains. **(D)** Bar plots showing enrichment analysis of ChromHMM annotation for H3K36me2 loss (left) and gain (right) in *Nsd2* KO E15.5 brains. Asterisks indicate significance (q value <0.05) for background *versus* H3K36me2 loss/gain using Fisher’s exact test.

To determine whether the loss of NSD2 affected H3K36me2 levels in the brain, we initially evaluated overall H3K36me2 levels. The bulk H3K36me2 levels were significantly decreased in E15.5 *Nsd2* KO brains (Supplementary Figure S3A). To gain more insight into the role of NSD2 in the H3K36me2 landscape in the brain, we conducted a ChIP-seq analysis using E15.5 brains. H3K36me2 ChIP-seq data were highly correlated (Spearman correlation coefficients >0.9) between biological replicates, and WT and *Nsd2* KO were separated into distinct clusters by hierarchical clustering (Supplementary Figure S3B). As the two biological replicates were well correlated across the genome, we merged them for further analysis. We identified 16.6% of the genome with H3K36me2 loss/gain in *Nsd2* KO brains (Figure 2A and Supplementary Tables S5, S6). Loss and gain of H3K36me2 were observed in both genic and intergenic regions (Figure 2B). Consistent with previous studies (Weinberg et al., 2019; Li et al., 2022), H3K36me2 loss was predominantly observed in the distal intergenic regions (Figure 2C). Conversely, the gain was largely observed in promoters and genic regions, including exons and intronic regions (Figure 2C).

To further characterize the regions where changes in H3K36me2 were observed, we applied ChromHMM annotations of developing mouse tissues from ENCODE (van der Velde et al., 2021) and performed enrichment analysis. The quiescent chromatin state (Quies), characterized by very low signals for canonical histone marks and high DNA methylation, accounted for a large fraction of H3K36me2 loss. H3K36me2 gain was enriched in actively transcribed genes (Tx and TxWk) and enhancer-related states (EnhG, Ehn, EnhLo, EnhPr, and EnhPois) (Figure 2D). Overall, NSD2-mediated H3K36me2 shows preferential enrichment at the quiescent genomic regions in the embryonic brain, and redistribution of H3K36me2 in *Nsd2* KO occurs mainly at actively transcribed genes and enhancers.

### 3.3 Changes in H3K36me2 affect gene expression related to synaptic transmission and formation in the brain

**FIGURE 3:**
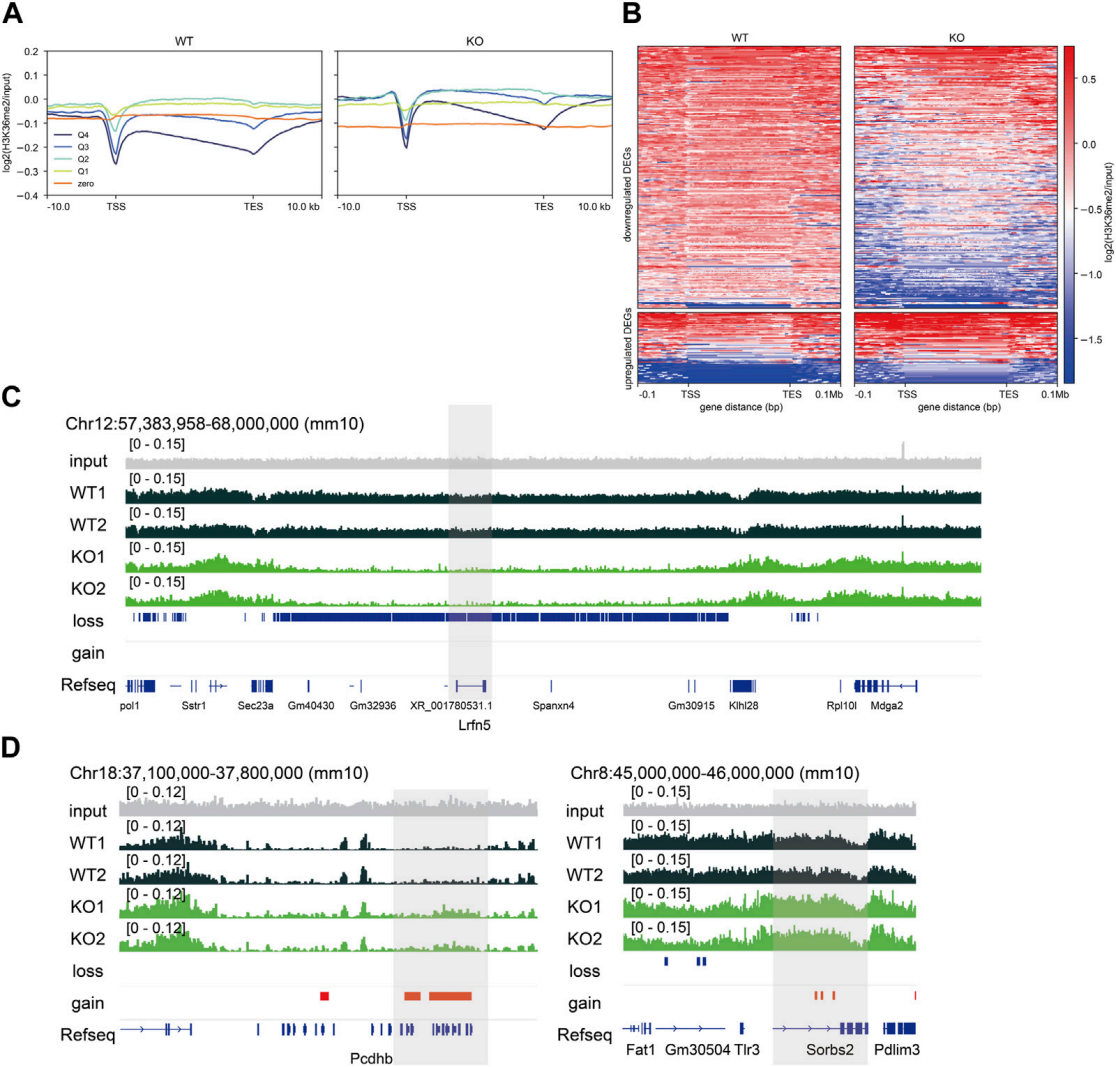
Relationship between gene expression and H3K36me2 levels. **(A)** Plot showing averaged H3K36me2 chromatin immunoprecipitation (ChIP) signal grouped into quartiles (Q4-Q1) and zero by transcripts per million and zero value in wild-type (WT) and knockout (KO) E15.5 brains. **(B)** Heatmaps showing H3K36me2 ChIP signal enrichment of differentially expressed genes (DEGs). **(C)** Genome browser snapshot showing representative H3K36me2 ChIP signal in WT and *Nsd2* knockout (KO) E15.5 brains. The shaded areas indicate *Lrfn5* (downregulated DEG) and **(D)** *Pcdhb* genes and *Sorbs2* (upregulated DEGs). H3K36me2 loss/gain in *Nsd2* KO are shown as red and blue bars, respectively, and genes (from RefSeq) are annotated at the bottom. TSS, transcription start site; TES, transcription end site.

Epigenetic states are associated with transcription, and their dysregulation causes transcriptional changes (Allis and Jenuwein, 2016). First, we examined the average H3K36me2 profile grouped by gene expression levels to characterize the relationship between H3K36me2 and gene expression levels. H3K36me2 profiles in highly expressed genes (Q4 and Q3) showed enrichment in the proximal region of the transcription start site (TSS) and gradually decayed in the 3′region of genes, consistent with previous studies (Kuo et al., 2011; Weinberg et al., 2019). In contrast, H3K36me2 profiles in weakly expressed genes and those with zero value (Q2, Q1, and zero) showed neutral patterns from the 5′to 3′region of genes except for the TSS with higher H3K36me2 levels than those of highly expressed genes (Figure 3A), suggesting that H3K36me2 levels are likely to be negatively correlated with gene expression levels in the embryonic brain. In accordance with the findings of the ChromHMM analysis, highly expressed genes (Q3, Q4) gained H3K36me2, while genes with zero value (zero) lost H3K36me2 in *Nsd2* KO.

Next, we examined whether changes in the levels of H3K36me2 were associated with changes in gene expression in *Nsd2* KO brains. We identified that 56% of DEGs showed changes in H3K36me2 levels (150/253 downregulated DEGs and 32/68 upregulated DEGs). The changes in H3K36me2 levels in upregulated DEGs were modest compared to the changes in downregulated DEGs. The majority of genes with H3K36me2 loss or gain showed no significant changes in gene expression (Supplementary Figure S4A). However, the DEGs with changes in H3K36me2 included genes related to synaptic transmission and formation. The downregulated or upregulated DEGs mainly showed loss or gain of H3K36me2 at their loci, respectively (Figures 3B–D), and we validated changes in gene expression and H3K36me2 using qPCR and ChIP-qPCR (Supplementary Figures S4B, S4C). Specifically, H3K36me2 loss in the downregulated DEGs was observed in a broader region extending up to 0.1 Mb upstream and downstream of TSS and transcription end site (TES) (Figures 3B, C). These results suggest that NSD2 affects the expression of genes important for neural function in the embryonic brain by regulating H3K36me2 at a sub-Mb scale domain.

H3K36me2 marks are typically located in both intergenic and genic regions. Changes in H3K36me2 at intergenic regions affect enhancer activity (Lhoumaud et al., 2019; Farhangdoost et al., 2021; Rajagopalan et al., 2021). To gain further insight into the functions of NSD2-mediated H3K36me2 in regulating gene expression, we examined the overlap between H3K36me2 loss and active enhancers. H3K36me2 loss included 183 active enhancers, defined by H3K4me1 and H3K27ac without H3K4me3 in E15.5 brain samples, but did not overlap with super-enhancers defined using H3K27ac and ROSE (Whyte et al., 2013). We identified 194 putative target gene active enhancer pairs, and these overlapped with only 54 DEGs (Supplementary Figure S4D). This indicates that changes in H3K36me2 may have a limited effect on enhancers in *Nsd2* KO.

## 4 Discussion

Perturbations in epigenetic regulation lead to NDDs, highlighting the importance of epigenetic modifiers in brain development and function. In this study, we characterized the transcriptomic and epigenomic changes in *Nsd2* KO brains using genome-wide approaches. Our analysis of transcriptomic profiles in *Nsd2* KO brains showed that the loss of NSD2 led to the dysregulation of gene expression involved in synaptic transmission and formation, which are essential for brain function. Synaptic dysfunction has been implicated in NDDs (Zoghbi and Bear, 2012; Südhof, 2018). The downregulated DEGs included genes previously reported to be associated with NDDs, such as *Gria4*, *Csmd3*, and *Kcnd2* (Lee et al., 2014; Martin et al., 2017; Lin et al., 2018; Zhang et al., 2021; Song et al., 2022; Wang et al., 2022). In addition, the upregulated DEGs included *Pcdhb* genes, which are involved in axon targeting and interneuron survival (Hasegawa et al., 2016). The upregulation of *Pcdhb* genes has been observed in several mouse models of NDD and ASD that exhibit abnormal behaviors (Balan et al., 2021; Hagelkruys et al., 2022). These observations suggest that the dysregulation of gene expression following the loss of NSD2 could contribute to neurological dysfunction. Further investigation is required to understand the connection between these gene expression changes and the pathogenesis of NDDs in *Nsd2* KO mice.

Although we observed a global loss of H3K36me2 in *Nsd2* KO brains, the loss of NSD2 did not completely deplete H3K36me2. Recent studies have reported that NSD1 is required for the patterning of H3K36me2 and DNA methylation in the nervous system and plays an important role in neuronal identity (Hamagami et al., 2023; Zheng et al., 2023). The loss of NSD1 in neocortices showed a global reduction in H3K36me2 throughout the whole genome, including both genic and intergenic regions. However, single knockouts of *Nsd1* or *Nsd2* result in limited loss of H3K36me2, suggesting that NSD1 and NSD2 redundantly contribute to the broad H3K36me2 pattern in the nervous system. Pathogenic variants of NSD1 have been identified in Sotos syndrome, which is characterized by overgrowth and learning disabilities (Kurotaki et al., 2002; Tatton-Brown and Rahman, 2007). In addition, pathogenic variants of the H3K36 methyltransferases SETD2 and ASH1L have also been identified in patients with NDDs (de Ligt et al., 2012; Luscan et al., 2014; Wang et al., 2016; Okamoto et al., 2017). Understanding the overlapping and distinct roles of these H3K36 methyltransferases will be useful for understanding the mechanism of related NDDs. Moreover, we also observed gains of H3K36me2 in *Nsd2* KO brains. Similar increases in H3K36me2 levels have also been observed in other knockout/knockdown experiments of H3K36 methyltransferases in mammalian cells (Weinberg et al., 2019; Farhangdoost et al., 2021; Hamagami et al., 2023). H3K36 methylation cross-talks with other epigenetic modifications, and changes in H3K36me2 can affect other histone modifications such as H3K27me3 and H3K27ac, DNA methylation, and chromatin accessibility (Streubel et al., 2018; Lhoumaud et al., 2019; Weinberg et al., 2019; Fang et al., 2021; Hamagami et al., 2023). Furthermore, since other H3K36 methyltransferases, including NSD1, SETD2, and ASH1L are also expressed in the developing brain, they can mediate H3K36me2 under conditions of NSD2 loss. Therefore, this might be a consequence of the redistribution of other H3K36 methyltransferases through changes in H3K36me2 and subsequent epigenetic modifications.

Using chromatin state annotation, it was found that NSD2-mediated H3K36me2 mainly marks quiescent state chromatin in the E15.5 brain. Additionally, higher H3K36me2 signals were observed in weakly expressed genes. Previous studies have shown that H3K36me2 is associated with actively transcribed genes in mammalian cells (Kuo et al., 2011; Weinberg et al., 2019). However, recent studies have suggested that impaired deposition of H3K36me2 leads to the loss of non-CpG methylation and gene activation in the adult mouse brain (Hamagami et al., 2023; Khazaei et al., 2023; Zheng et al., 2023). Non-CpG methylation in the brain is acquired during postnatal development (Lister et al., 2013). Therefore, H3K36me2 may serve as a bookmark for gene repression in the embryonic brain.

In our study, we observed changes in H3K36me2 in a subset of DEGs, suggesting that these genes are direct targets of NSD2. On the other hand, there are DEGs that were not affected by H3K36me2 in *Nsd2* KO. Histone modification enzymes, including NSD2, have non-catalytic functions (Tian et al., 2019; Sun et al., 2023). These proteins bind to chromatin and act as transcriptional coregulators independently of their catalytic activity. Additionally, NSD2 methylates non-histone proteins (Park et al., 2018; Zhang et al., 2019). Non-histone methylation has been implicated in protein-protein interactions and affects the downstream pathway of the substrate protein. Such non-canonical functions of NSD2 may explain DEGs that are not affected by changes in H3K36me2. Additionally, most genes with loss of H3K36me2 did not show changes in expression or enrichment of any GO terms or KEGG pathways. These included clusters of genes, such as olfactory receptor genes, keratin genes, and immune genes (Supplementary Table S6). Further investigation into the detailed mechanisms of NSD2 recruitment and gene regulation by NSD2 in the brain is warranted.

The present study has limitations that need to be considered. We used bulk embryonic brain tissue composed of heterogeneous cell populations. This was limited to accurately assessing changes in gene expression and H3K36me2 levels. Furthermore, we focused on the brain at the embryonic stage due to high levels of *Nsd2* expression. The development and maturation of the mammalian brain continue after birth with epigenetic changes (Lister et al., 2013). Dynamic analysis of specific cell types across brain development can provide deeper insights into NSD2 function in the brain.

In summary, our results showed that NSD2 is involved in gene regulation that is important for neural functioning through H3K36me2. Elucidating the function of NSD2 in the brain is important for understanding NSD2-associated NDDs and epigenetic regulation in the brain.

## Data Availability

The datasets presented in this study can be found in online repositories. The names of the repository/repositories and accession number(s) can be found below: https://www.ncbi.nlm.nih.gov/geo/, GSE232566.

## Footnotes

1
http://hannonlab.cshl.edu/fastx_toolkit/

2
https://broadinstitute.github.io/picard/
