# Supplementary material for: Loss of NSD2 causes dysregulation of synaptic genes and altered H3K36 dimethylation in mice
Source: Front Genet. 2024 Feb 14;15:1308234. doi: 10.3389/fgene.2024.1308234 (PMC10899350; doi:10.3389/fgene.2024.1308234)
Supplement: Supplementary file 3 [file Image5.PDF]

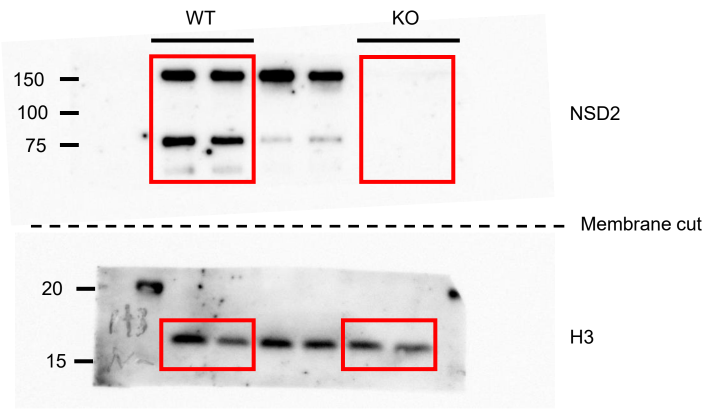

Supplementary Figure S5. Uncropped western blotting image. An uncropped version of the western blotting image presented in Supplementary Figure 1B. The red frames represent the cropped area
