# Supplementary material for: Loss of NSD2 causes dysregulation of synaptic genes and altered H3K36 dimethylation in mice
Source: Front Genet. 2024 Feb 14;15:1308234. doi: 10.3389/fgene.2024.1308234 (PMC10899350; doi:10.3389/fgene.2024.1308234)
Supplement: Supplementary file 4 [file Image4.PDF]

A

gene expression	H3K36me2 loss	H3K36me2 gain
down	144	6
unchanged	6675	2806
up	1	31

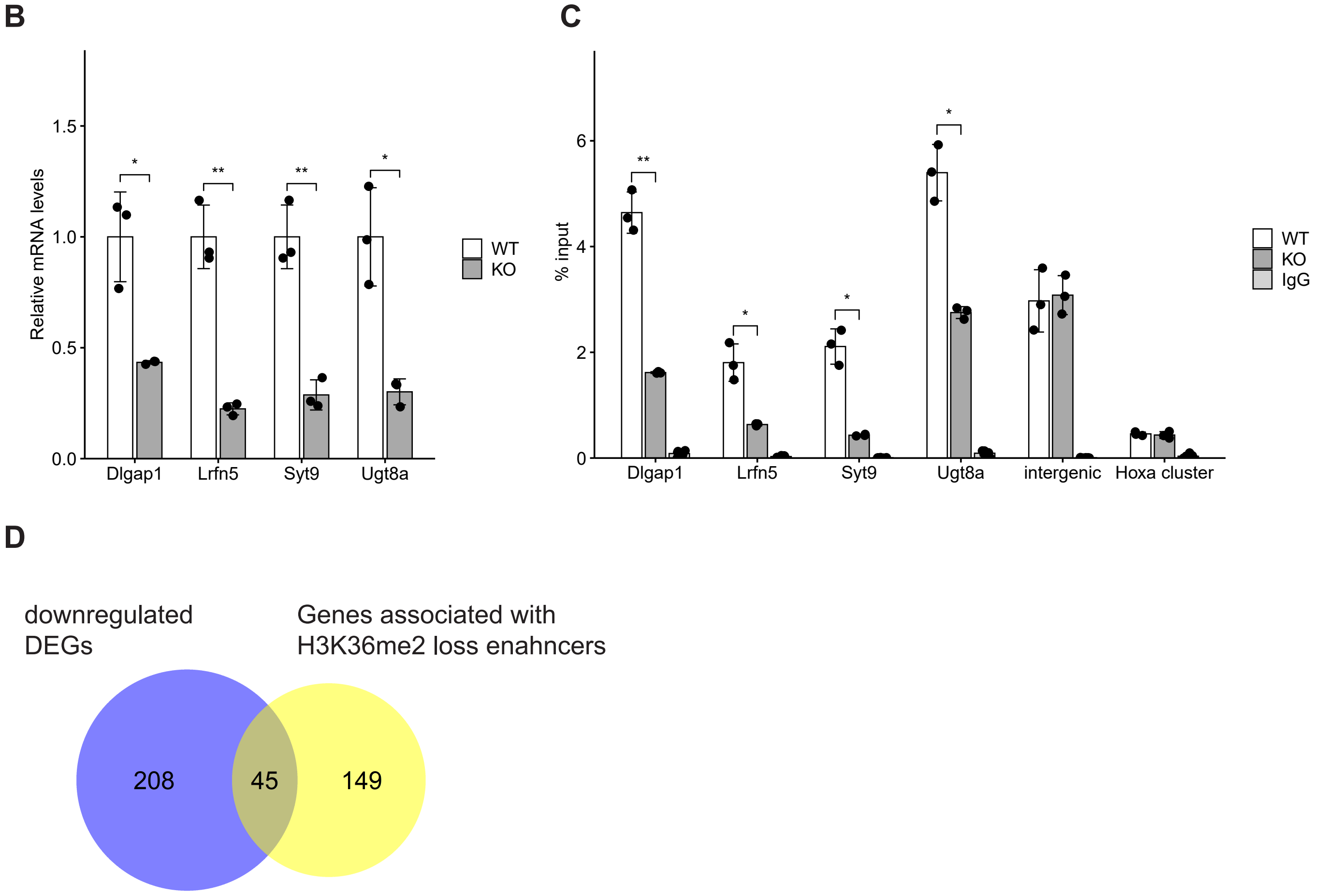

Supplementary Figure S4. Changes in transcription and H3K36me2 in *Nsd2* knockout (KO) brains. (**A**) Table summarizing the results of changes in H3K36me2 at gene loci and changes in gene expression. The numbers represent the number of genes in each category. (**B**) RT-qPCR of representative synaptic function-related genes with H3K36me2 loss in wild-type (WT) and *Nsd2* KO brains. Data are presented as mean ± SD with dots representing individual samples; WT (n = 3; male = 2, female =1), KO (n = 3; male = 3). *$p < 0.05$, **$p < 0.01$, calculated using Welch's *t*-test. (**C**) H3K36me2 ChIP-qPCR of representative synaptic function-related genes with H3K36me2 loss in WT and *Nsd2* KO brains. The input was used for normalization. Intergenic region and Hoxa cluster were used as positive and negative control regions of H3K36me2 ChIP, respectively. Data are shown as mean ± SD with dots representing individual samples; WT (n = 3; male =1, female = 2), KO (n = 3; male = 2, female = 1), IgG (n = 6). *$p < 0.05$, **$p < 0.01$, calculated using Welch's *t*-test. (**D**) Venn diagram showing the overlap between differentially expressed genes and genes associated with H3K36me2 loss enhancers in *Nsd2* KO.
