# Supplementary material for: Loss of NSD2 causes dysregulation of synaptic genes and altered H3K36 dimethylation in mice
Source: Front Genet. 2024 Feb 14;15:1308234. doi: 10.3389/fgene.2024.1308234 (PMC10899350; doi:10.3389/fgene.2024.1308234)
Supplement: Supplementary file 5 [file Image2.PDF]

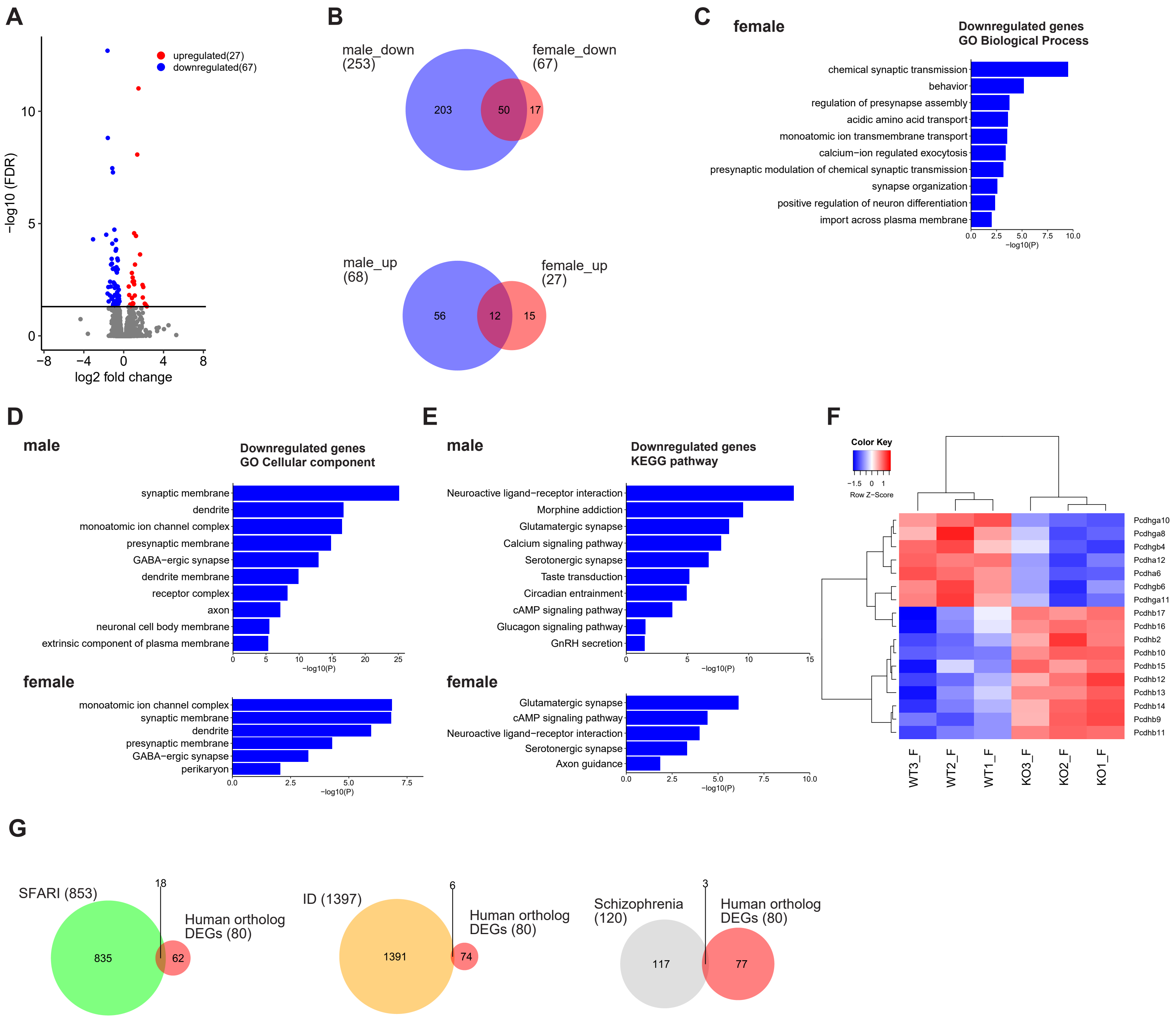

Supplementary Figure S2. RNA sequencing analysis of *Nsd2* knockout (KO) brains. (**A**) Volcano plot of RNA sequencing analysis showing differentially expressed genes (DEGs) between *Nsd2* KO and wild-type (WT) female mouse brains at E15.5; n = 3 in each group. DEGs are labeled in blue (downregulated) and red (upregulated). The horizontal line indicates the false discovery rate (FDR) threshold of 0.05. (**B**) Venn diagram demonstrating an overlap between male and female downregulated DEGs (top) and upregulated DEGs (bottom). **(C to E)** Bar plots showing enriched Gene Ontology terms among biological processes in *Nsd2* KO female (C), cellular components (D) and enriched Kyoto Encyclopedia of Genes and Genomes pathway (E) in *Nsd2* KO males and females. (**F**) Heatmap showing clustered protocadherin genes that are differentially expressed in *Nsd2* KO females by hierarchical clustering of WT and *Nsd2* KO biological replicates. (**G**) Venn diagram showing the overlap between human ortholog DEGs in *Nsd2* KO females and neurodevelopmental disorder gene sets, including autism spectrum disorder from SFARI (left), intellectual disability (middle), and schizophrenia (right). ASD, autism spectrum disorder; ID, intellectual disability.
