# Supplementary material for: Loss of NSD2 causes dysregulation of synaptic genes and altered H3K36 dimethylation in mice
Source: Front Genet. 2024 Feb 14;15:1308234. doi: 10.3389/fgene.2024.1308234 (PMC10899350; doi:10.3389/fgene.2024.1308234)
Supplement: Supplementary file 7 [file Image3.PDF]

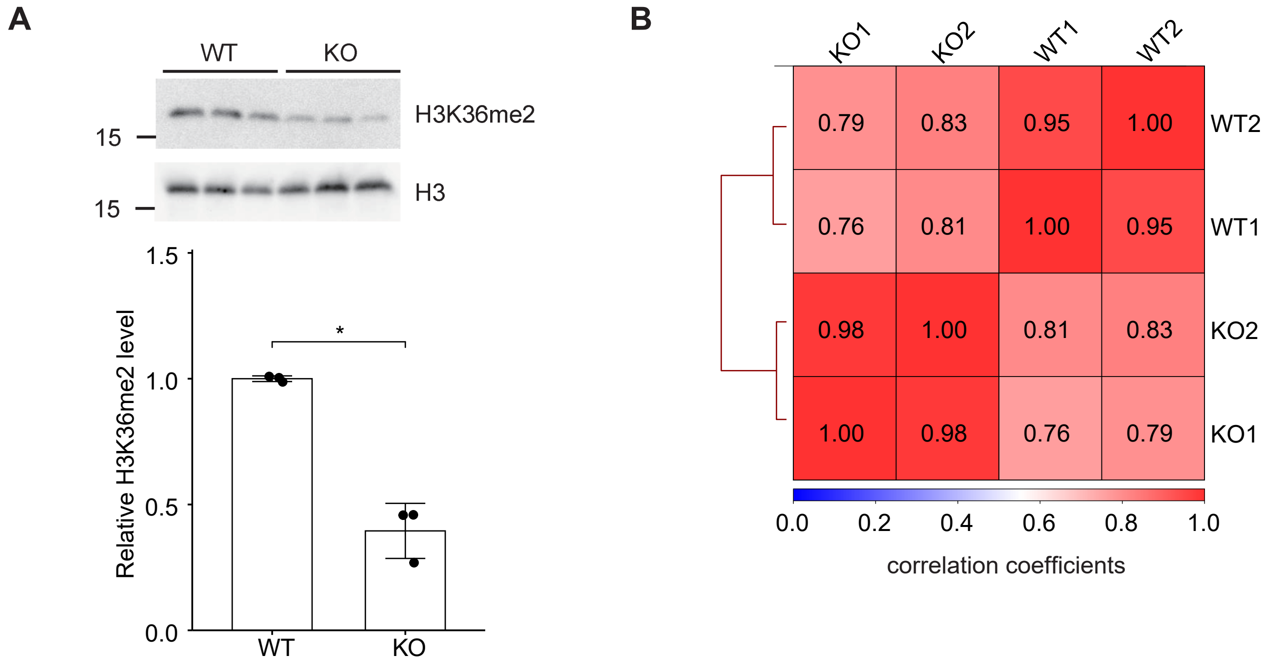

Supplementary Figure S3. H3K36me2 chromatin immunoprecipitation sequencing analysis in *Nsd2* knockout (KO) brains. (**A**) Western blotting of H3K36me2 in E15.5 wild-type (WT) and *Nsd2* KO mouse brains. Histone H3 was used as a loading control. The signal intensity values were divided by those of H3 and plotted relative to the control. Data are presented as mean ± SD with dots representing individual samples; n = 3 in each group. *$p < 0.05$, calculated using Welch's t-test. (**B**) The heatmap demonstrates Spearman's correlation and hierarchical analysis of H3K36me2 chromatin immunoprecipitation sequencing data; n = 2 in each group. Correlation coefficients are depicted by color intensities and numbers.
