# Supplementary material for: Loss of NSD2 causes dysregulation of synaptic genes and altered H3K36 dimethylation in mice
Source: Front Genet. 2024 Feb 14;15:1308234. doi: 10.3389/fgene.2024.1308234 (PMC10899350; doi:10.3389/fgene.2024.1308234)
Supplement: Supplementary file 11 [file Image1.pdf]

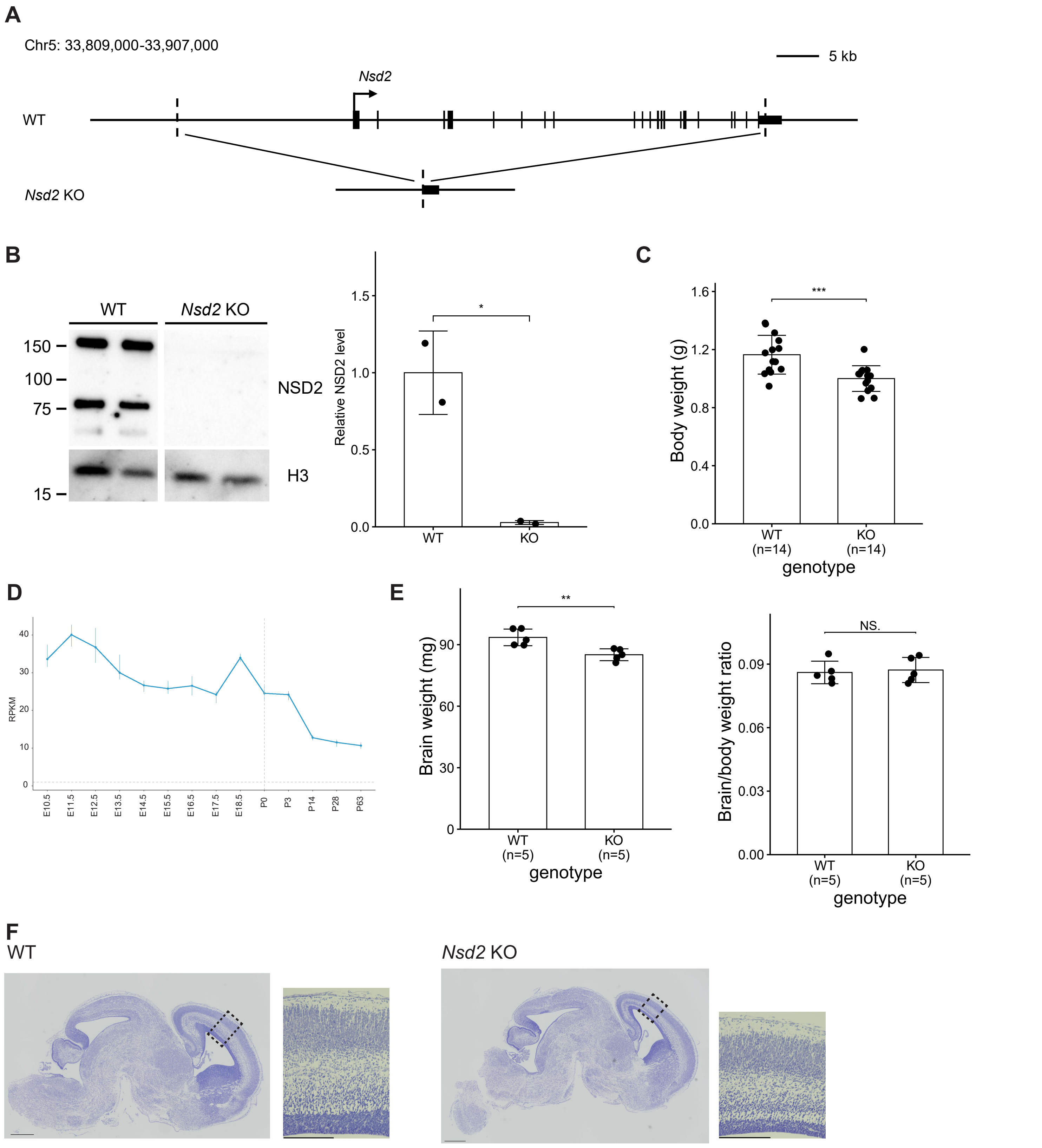

Supplementary Figure S1. *Nsd2* knockout (KO) mice. (**A**) Schematic representation of wild-type (WT) and *Nsd2* KO alleles. The black boxes represent the exons. The dashed lines indicate guide RNA targeting sites for CRISPR/Cas9. (**B**) Western blotting of NSD2 in WT and *Nsd2* KO brains. Histone H3 was used as a loading control. The values of signal intensity were divided by that of H3 and normalized to the control. Data are shown as mean ± SD with dots representing individual samples; n = 2 in each group. *$p < 0.05$, calculated using Student's t-test. The uncropped image of western blotting is provided in Supplementary Figure S5. (**C**) Bar plot showing the body weight of WT compared to *Nsd2* KO embryos at E18.5. Data are shown as mean ± SD with dots representing individual samples; n = 14 in each group. ***, $p < 0.001$, calculated using Student's t-test. (**D**) The graph shows the expression pattern of *Nsd2* in the brain during mouse development; data were obtained from the research by Cardoso-Moreira *et al.* (**E**) Bar plot showing brain weights (left) and brain/body ratio (right) of WT compared to *Nsd2* KO embryos at E18.5. Data are shown as mean ± SD with dots representing individual samples; n = 5 in each group. **$p < 0.01$, NS., not significant, calculated using Student's t-test. (**F**) Representative histological images of Nissl staining in E18.5 WT (left) and *Nsd2* KO (right) sagittal brain sections. The boxed regions were magnified on the right. Scale bars, 500 μm and 200 μm (magnified images).
